# Defining New Research Questions and Protocols in the Field of Traumatic Brain Injury through Public Engagement: Preliminary Results and Review of the Literature

**DOI:** 10.1155/2019/9101235

**Published:** 2019-10-31

**Authors:** Shumaila Hasan, Aswin Chari, Mario Ganau, Chris Uff

**Affiliations:** ^1^Department of Neurosurgery, The Royal London Hospital, London E1 1BB, UK; ^2^Department of Neurosurgery, Oxford University Hospitals, Oxford OX3 9DU, UK

## Abstract

Traumatic brain injury (TBI) is the most common cause of death and disability in the age group below 40 years. The financial cost of loss of earnings and medical care presents a massive burden to family, society, social care, and healthcare, the cost of which is estimated at £1 billion per annum (about brain injury (online)). At present, we still lack a full understanding on the pathophysiology of TBI, and biomarkers represent the next frontier of breakthrough discoveries. Unfortunately, many tenets limit their widespread adoption. Brain tissue sampling is the mainstay of diagnosis in neuro-oncology; following on this path, we hypothesise that information gleaned from neural tissue samples obtained in TBI patients upon hospital admission may correlate with outcome data in TBI patients, enabling an early, accurate, and more comprehensive pathological classification, with the intent of guiding treatment and future research. We proposed various methods of tissue sampling at opportunistic times: two methods rely on a dedicated sample being taken; the remainder relies on tissue that would otherwise be discarded. To gauge acceptance of this, and as per the guidelines set out by the National Research Ethics Service, we conducted a survey of TBI and non-TBI patients admitted to our Trauma ward and their families. 100 responses were collected between December 2017 and July 2018, incorporating two redesigns in response to patient feedback. 75.0% of respondents said that they would consent to a brain biopsy performed at the time of insertion of an intracranial pressure (ICP) bolt. 7.0% replied negatively and 18.0% did not know. 70.0% would consent to insertion of a jugular bulb catheter to obtain paired intracranial venous samples and peripheral samples for analysis of biomarkers. Over 94.0% would consent to neural tissue from ICP probes, external ventricular drains (EVD), and lumbar drains (LD) to be salvaged, and 95.0% would consent to intraoperative samples for further analysis.

## 1. Introduction

Traumatic brain injury (TBI) is the most common cause of death and disability in the age group below 40 years. 1.4 million people attend Emergency Departments (ED) with a recent head injury annually in England and Wales alone [1]. TBI is a significant public health concern and has been estimated to contribute up to 41% of overall years of life lost (YLL) due to injuries [2]. In addition to the direct impact, there is a significant indirect impact on need for assistance with self-care, employment productivity, and social relationships, especially given that the majority of those with severe TBI are young adults [3]. Primary injury occurs at the time of impact, whereas secondary injury, also termed “delayed nonmechanical damage,” occurs due to disruption of the normal metabolism often resulting in inflammation and necrosis. Primary injury cannot be modulated once the injury has occurred, but the focus of TBI care is on preventing or attenuating secondary injury.

The 2016 Brain Trauma Foundation Guidelines [4] advocate management of severe diffuse TBI conservatively in Intensive Care Units (ICU) with neuroprotective measures, guided by intracranial pressure (ICP) monitoring, with the potential to provide cerebrospinal fluid (CSF) drainage by inserting an external ventricular drain (EVD) if the ICP remains high. In cases where there is a haematoma amenable to surgical evacuation, patients may undergo a craniotomy or craniectomy to decompress the brain. Thus, current therapeutic options are limited to relieving pressure (by evacuating haematomas, removing bone, or draining CSF) and supporting the patient with ICU care [5]. Despite many trials there are still no therapeutic options which alter the TBI, and treatment centres on supporting the patient while the brain injury runs its course.

Although progress has been made in unpicking the pathophysiology of TBI [6], this has not translated into clinical practice. Despite significant research effort, there are currently no therapeutic options, other than decompression and support, which actively alter the course of the injury. Numerous studies into biomarkers released in the acute phase have been conducted [7–27]; however, it is important to note that these samples are obtained peripherally, and we have no knowledge of how much information is lost when brain venous blood mixes with systemic venous blood.

S100B [7, 11, 12] is perhaps the most extensively studied biomarker in TBI; however, it has been found elevated in patients with polytrauma suggesting concentrations are affected by extracerebral injuries. Galectin-3 (GAL3) [8, 21] is a proinflammatory protein expressed during inflammation of the central neurological system, with a positive correlation being identified between plasma concentrations of GAL3 and Glasgow Coma Scale (GCS) scores, suggesting it may reflect trauma severity. Copeptin [23, 26] levels also show correlation with poor outcomes, and elevated levels of neuron-specific enolase (NSE) [7, 13, 14] have been demonstrated to be an indicator of mortality. The combination of ubiquitin C-terminal hydrolase L1 (UCH-L1) [9, 22, 27] and glial fibrillary acidic protein (GFAP) [10, 22] have been shown to produce superior sensitivity and specificity when distinguishing patients with TBI from healthy controls. Release of matrix metalloproteinase 9 (MMP9) [8] can be found up to 8 hours after mild TBI, whereas myelin basic protein (MBP) [7, 15, 16] often peaks between 48 and 72 hours after injury and can remain elevated for up to 2 weeks. MBP is also considered to be a potential biomarker of intracranial haemorrhage and traumatic axonal injury [18], and MBP along with myelin-associated glycoprotein (MAG) are products of oligodendrocyte demyelination which can be predictive of functional outcomes in patients with mild TBI [17]. The measurement of tau protein in CSF in patients who have sustained severe TBI has a proven correlation with outcome; however, it is a poor predictor when measured in peripheral blood or in mild TBI [24]. As demonstrated by this brief summary on the state of the art of TBI-specific biomarkers, the research interest into this area of neurotrauma is on the rise specifically for the expected leap forwards in terms of understanding of the physiopathology and possibility to design much sought after prognostication tools (see Table 1).

Animal models analysing the microscopic and cellular effects of TBI [28–30] have been conducted; however, these have limited application because they do not necessarily translate accurately to humans and human data remain scarce [31]. Brain tissue sampling outside of tumour surgeries has been done very occasionally, with Harish et al. [32] obtaining intraoperative open biopsy samples from 26 patients who had sustained TBI and correlating them to CT scans and Pyykkö et al. [33] obtaining cortical brain biopsies using a biopsy needle from 102 patients with normal pressure hydrocephalus (NPH).

For these reasons, among others, prognosis in TBI remains extremely difficult. Various models exist such as the International Mission for Prognosis and Analysis of Clinical Trials (IMPACT) trial in TBI [34]; however, their reliability in predicting an unsurvivable injury or a very poor neurological outcome is not high enough to permit withdrawal of care. Although the abovementioned biomarkers bring new hope, further research into their sensitivity and specificity is needed to appraise their possible role in clinical practice. As such, current management still revolves solely around prolonged ITU care and rehabilitation with significant resource implications which not infrequently result in patients left in a vegetative or minimally conscious state.

The authors propose a novel prospective study to obtain fresh brain tissue samples from patients suffering severe TBI, in order to correlate the cellular, proteomic, metabolomic, and, in the future, genomic data to clinical outcome. We also propose obtaining pure brain venous blood from jugular bulb venous catheters to analyse brain venous biomarkers and compare them to paired peripheral samples.

Patients suffering severe TBI will, by definition, not be able to consent to participate in research, and often, next of kin are often not immediately available. According to the guidelines set out by the National Research Ethics Service, we conducted a community consultation survey of patients and their families in order to establish their perception of what we propose.

## 2. Methods

A pilot survey was created and administered to 26 patients and families of patients who had been treated at our Institution for traumatic brain injury. This survey was registered as a service evaluation and obtained ethical approval by our Institutional Review Board. It was conducted at the Royal London Hospital, which serves a population of >5mln people; it represents the busiest Major Trauma Centre in London and accounts among the biggest neurotrauma hubs in the United Kingdom. The aim of the survey was to gauge public perception of whether taking samples that would usually be discarded for further analysis was acceptable. We included the question of collecting additional blood tests and inserting a jugular bulb venous catheter to collect venous drainage from the brain.

Feedback gathered from the pilot survey was used to create a further survey (Survey 1), containing more information. We introduced the question of obtaining a brain biopsy at the time of insertion of an ICP bolt and urine, saliva, and stool samples taken concurrently for evaluation of biomarkers.

Survey 1 was administered to four groups of patients from our hospital and its catching area (East London): patients suffering head injury and their family members on neurosurgical wards; patients attending the TBI follow-up clinic; a population of previous general trauma patients who had expressed an interest in assisting with research questions using Survey Monkey; TBI patients attending the East London Headway (a UK-based brain injury charity) centre. All responses were anonymised. All respondents had the capacity to fill out the questionnaire unaided. Apart from the Survey Monkey group, all participants had the opportunity to ask for additional information, which was provided by the staff administering the survey if required.

Feedback from Survey 1 regarding the anonymity and storage of samples was incorporated into a further version of the survey (Survey 2) to address those concerns and provide additional information. Overall, respondents had more understanding following these changes and seemed more satisfied with the amount of information provided.

When analysing the data, the pilot survey figures were considered separate to those of the main survey. The responses to Survey 1, Survey 2, and Survey Monkey responses were collated and analysed.

The pilot survey, Survey 1, and Survey 2 are attached as supplementary materials ([Supplementary-material supplementary-material-1]).

## 3. Results

**Figure 1:**
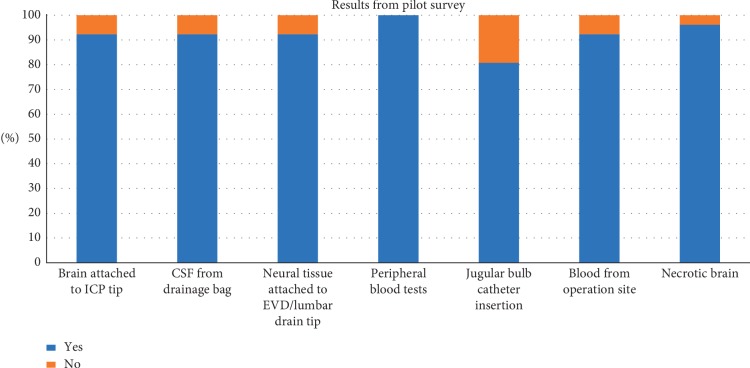
Results from the pilot survey.

The pilot survey demonstrated that 92.3% of respondents were willing to support research on the neural tissue adherent to ICP probes and EVD/LD requiring appropriate storage and analyses meant to identify cellular and molecular changes. Furthermore, 96.2% of respondents were willing to support research on necrotic brain tissue resected at the time of surgery for the same purposes, and 80.8% were willing for a jugular bulb catheter to be inserted to ascertain the biomarker load directly from the venous drainage of the brain (see Table 2, and Figure 1).

After updating the survey, a further 100 responses were collected. The results show that only 7.0% of respondents would not consent to brain biopsy at the time of ICP bolt insertion, whereas 75.0% would agree to brain biopsy at time of ICP bolt and 18.0% declared to be unsure. This distribution highlights how difficult it may be for the general population to understand these concepts, particularly in online surveys where the opportunity to ask further questions is restricted. Additionally, the 18 “do not know” respondents to brain biopsy, 7 “no's” and 75 “yes's,” although not posing any conceptual issue of our surveys, may demonstrate the potential indecisiveness of people dealing with an immediate decision in an acute trauma situation.

**Figure 2:**
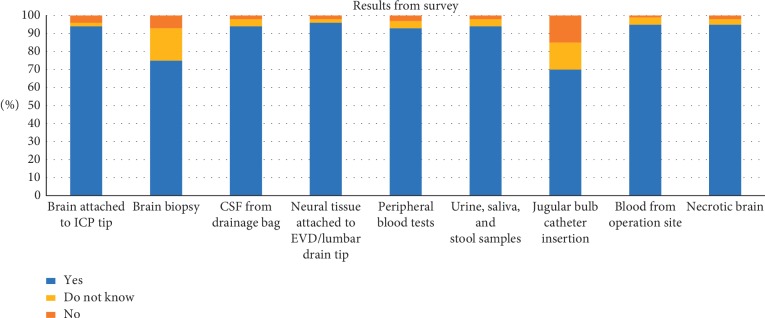
Results from the survey.

This said, our surveys indicate that 94.0% would consent to brain tissue adherent to ICP probes and 96.0% would consent to neural tissue adherent to EVD and LD tips, to be used for further analysis. Of note, 95.0% would agree to have intraoperative samples being taken, both from blood at the operating site and necrotic brain that would otherwise be discarded, and 70.0% would agree to a jugular bulb catheter to be inserted for obtaining venous samples from the brain (see Table 3, and Figure 2).

## 4. Discussion

This community consultation has revealed an overwhelmingly positive opinion among TBI patients and their families regarding the prospect of further research into TBI, particularly with respect to obtaining brain biopsies at the time of insertion of ICP monitors, insertion of jugular bulb catheters, and using brain tissue and CSF samples that would otherwise be discarded. Patients with severe TBI are incapacitated due to the nature of the TBI and are thus unable to consent for themselves; therefore, public and patient surveys are vital as they explore the surrounding ethical issues and gauge acceptance of the proposed research. We chose not to involve families of nonsurvivors of TBI to eliminate the emotional bias that may be encountered in their responses when compared to rational answers.

The support and enthusiasm we received during the course of this community consultation highlight the recognition from people who have been affected by TBI that further work is required in understanding the physiopathology of TBI, thereby potentially improving prognostication and furthering treatment strategies.

Although the question of surrogate consent was not included in our survey, this is dealt with specifically by the 2005 Mental Capacity Act (sections 30–33) which provides lawful authority for intrusive research to be carried out involving people without capacity provided that the research has been approved by an appropriate body. Previous community consultations in emergency neurosurgical procedures in incapacitated patients have shown this is an acceptable method of inclusion in studies involving these patients [35–37]. While this is a good starting point, we should highlight that each of those studies related to the use of potential treatment that may have had direct benefit to the subject. In fact, the Corticoid Randomization after Significant Head Injury (CRASH) Trial [35] evaluated tranexamic acid in TBI; Clark et al. [36] was evaluating the role of decompressive craniectomy for evacuation of an acute subdural haematoma, and Scotton et al. [37] was evaluating a subdural evacuating port system as a minimally invasive alternative to burr-hole evacuation. On the contrary, the study outlined in our surveys, although not influencing our standard of care, might not necessary lead to any direct benefit for the participants. For this very reason, community consultation are considered prior to starting such type of investigational clinical trial, where the patient is unlikely to have capacity to consent to enrolment in the trial, but the proposed brain biopsy is for exploratory biomarker research that would not have a potential direct benefit to that subject.

There are many proposed hypotheses regarding the pathophysiology behind TBI; however, there remains limited information, and there are suggestions of increasing inpatient mortality rates following TBI over the last three decades [38, 39]. The reasons for this are multifactorial with many attributing the reason for the lack of improvements being made in the field to the lack of understanding of the underlying mechanisms and the restriction to experimental models [32]. In addition, prognostication remains a significant challenge as previously pointed out [41] despite the models proposed by the IMPACT and CRASH trials mentioned above.

Harish et al. [32] obtained brain tissue from 26 patients who had sustained TBI and underwent craniotomy and found distinct alterations specific to contusions and pericontusions at both the tissue and cellular levels in one of the first studies to investigate anatomical, cellular, and molecular changes in human TBI. Among their key findings was that pericontusional areas were susceptible to cytotoxic factors released by contusions, offering a window for repair and neuroregeneration and implying therapeutic options may be available. We believe that this study is crucial and opens the possibility for further work in human TBI, which is likely to provide more relevant information than histopathological research into animal models or postmortem studies.

Pyykkö et al. [33] obtained cortical brain biopsies from 102 patients with NPH and investigated the association between proinflammatory cytokines and biomarkers of neuronal damage in CSF. This study sets a precedent for obtaining *in vivo* brain tissue samples via needle biopsy outside the realm of brain tumour surgery. As the current study demonstrates, this concept is not straight-forward; however, only 7% stated that they would not consent to this.

Techniques such as microdialysis for the sampling of biomarkers in CSF interstitial space are well established, however limited in their application due to the difficulty in effectively estimating the concentration of the protein of interest *in vivo* [41]. Further work into point-of-care diagnostics has greatly enhanced the possibility of quick and inexpensive methods of detecting proteins of interest, and these tests can be performed in the emergency department or at the bedside. Besides saliva and plasma, attention is being given to CSF to show meaningful changes or imbalances in neural tissues [17].

Biomarkers in TBI have been gathering increasing attention; however, the majority of published studies focus on peripheral blood samples. Insights on biomarker trends after TBI through samples obtained from pure brain venous blood (obtained from the jugular venous bulb) paired with those obtained from peripheral blood may prove useful to understand how much information is lost in peripheral venous blood, as proven in previous pilot studies [42]. In addition, this may permit correlation between the two and allow validation of peripheral samples.

## 5. Limitations of This Current Study

Responders to these surveys included patients who had previously sustained TBI and their families; an element of bias may therefore be present. In addition, although all respondents were able to complete the survey unaided, information pertaining to their education, intellectual capacity, and the degree of recovery was not recorded nor subject to further analysis.

Performing the pilot survey was important for the tool development and orientating the further steps in designing Survey 1 and improving it with Survey 2. Gathering all the data acquired in the course of this community consultation and conducting a final analysing can be criticized because of the slight difference of information received by participants at different stages and different methods of collection. Even though this has to be recognized as a limitation of the study, we considered this choice methodologically sounding because the questions asked in the surveys were basically very similar, if not identical.

## 6. Conclusion

Current management of TBI relies on decompression of salvageable brain and supportive care. There are currently no available treatments which aim to attenuate secondary injury. We hypothesise that the information gained from *in vivo* tissue sampling may direct future research into more accurate prognostic models and therapeutic options. As part of the research protocol, we conducted a community consultation aimed at investigating patients' understanding of the research challenges and their support toward future studies. The responses collected were overall very favourable, with many patients and their families recognising the utility and importance of this public engagement project and an overwhelming percentage willing to consent for samples being taken for this purpose in case of future studies. The responses to these surveys are particularly relevant because they suggest a wide community consensus in support of this type of research, hence partly answering the ethical questions revolving around participation of patients who have sustained severe TBI and therefore incapacitated and unable to consent to participation in research. Given the above, we hope that our study will contribute to provide evidence in support of research protocols requiring *in vivo* tissue sampling from patients suffering severe TBI.

## Figures and Tables

**Table 1:** Biomarkers and their applications.

Biomarker	Key features	References
S100B	Serum concentrations >0.48 *μ*g/L in <6 hours predictive of Glasgow Outcome Scale Extended (GOSE) scores of <5 (severe disability) at 1 month post injury Extracerebral injuries have significant impact on predictive ability of S100B	[7, 11, 12]

GAL3	High plasma levels associated with GCS and in-hospital mortality	[8, 21]

Copeptin	Independent predictor of progressive haemorrhagic injury and acute traumatic coagulopathy and outcome at 1 year after injury	[23, 26]

NSE	>10 *μ*g/L in <6 hours associated with headaches at 6 months Elevated levels indicator of mortality	[7, 13, 14]

UCH-L1	Plasma and CSF levels shown to be elevated for several days and associated with diffuse injuries	[9, 22, 27]

GFAP	Elevations primarily found in patients with a focal mass lesion (V to Marshall VI) When Marshall is combined with GFAP, it produces superior sensitivity and specificity for TBI	[10, 22]

MMP9	Elevated levels up to 8 hours after TBI; smaller increase maintained at 24 hours	[8]

MBP	Serum concentrations peak 48–72 hours after injury and can remain elevated for 2 weeks Potential biomarker of intracranial haemorrhage and axonal injury	[7, 15, 16, 18]

MAG	Strong predictors of functional outcome in mild TBI	[17]

Tau	Raised CSF levels associated with poor clinical outcome	[24]

**Table 2:** Results from pilot survey.

Question		Answers (*n*)	%
Brain attached to ICP tip	Yes	24	92.3
	No	2	7.7
CSF from drainage bag	Yes	24	92.3
	No	2	7.7
Neural tissue attached to EVD/lumbar drain tip	Yes	24	92.3
	No	2	7.7
Peripheral blood tests	Yes	26	100
	No	0	0
Jugular bulb catheter insertion	Yes	21	80.8
	No	5	19.2
Blood from operation site	Yes	24	92.3
	No	2	7.7
Necrotic brain	Yes	25	96.2
	No	1	3.8

**Table 3:** Results from survey.

Question					Total (*n*)	%
		Survey 1	Survey Monkey	Survey 2		
Brain attached to ICP tip	Yes	33	28	33	94	94
	No	0	1	3	4	4
	Do not know	2	0	0	2	2

Brain biopsy	Yes	27	17	31	75	75
	No	2	2	3	7	7
	Do not know	6	10	2	18	18

CSF from drainage bag	Yes	32	29	33	94	94
	No	0	0	2	2	2
	Do not know	3	0	1	4	4

Neural tissue attached to EVD/lumbar drain tip	Yes	33	29	34	96	96
	No	0	0	2	2	2
	Do not know	2	0	0	2	2

Peripheral blood tests	Yes	33	27	33	93	93
	No	0	0	3	3	3
	Do not know	2	2	0	4	4

Urine, saliva, stool samples	Yes	33	27	34	94	94
	No	1	0	1	2	2
	Do not know	1	2	1	4	4

Jugular bulb catheter insertion	Yes	25	17	28	70	70
	No	7	3	5	15	15
	Do not know	3	9	3	15	15

Blood from operation site	Yes	33	29	33	95	95
	No	0	0	1	1	1
	Do not know	2	0	2	4	4

Necrotic brain	Yes	34	29	32	95	95
	No	0	0	2	2	2
	Do not know	1	0	2	3	3

## Data Availability

The questionnaire data used to support the findings of this study are available from the corresponding author upon request.

## Supplementary Materials

Supplementary Materials Supplementary material 1: pilot survey created to gauge response from head injury patients and their families to establish perceived acceptability to conducting laboratory analysis of neural tissue that may otherwise be discarded, obtaining peripheral blood samples, and for the insertion of jugular bulb catheters in patients with severe traumatic brain injuries. Supplementary material 2: Survey 1 created following feedback from the pilot survey. This contained further details and the response option of “do not know.” This also included new questions regarding obtaining additional tissue, i.e., brain biopsies at the time of insertion of intracranial pressure monitors, and obtaining extra samples of bodily fluids for analysis. Supplementary material 3: Survey 2 created following feedback from Survey 1 to answer questions regarding anonymity, the secure storage of samples in our laboratory, and that no further research investigations would be conducted at follow-up.
