# Supplementary material for: Defining New Research Questions and Protocols in the Field of Traumatic Brain Injury through Public Engagement: Preliminary Results and Review of the Literature
Source: Emerg Med Int. 2019 Oct 31;2019:9101235. doi: 10.1155/2019/9101235 (PMC6875310; doi:10.1155/2019/9101235)
Supplement: Supplementary Materials — Supplementary material 1: pilot survey created to gauge response from head injury patients and their families to establish perceived acceptability to conducting laboratory analysis of neural tissue that may otherwise be discarded, obtaining peripheral blood samples, and for the insertion of jugular bulb catheters in patients with severe traumatic brain injuries. Supplementary material 2: Survey 1 created following feedback from the pilot survey. This contained further details and the response option of “do not know.” This also included new questions regarding obtaining additional tissue, i.e., brain biopsies at the time of insertion of intracranial pressure monitors, and obtaining extra samples of bodily fluids for analysis. Supplementary material 3: Survey 2 created following feedback from Survey 1 to answer questions regarding anonymity, the secure storage of samples in our laboratory, and that no further research investigations would be conducted at follow-up. [file 9101235.f1.zip › 9101235.f1/Pilot survey.docx]

The Royal London Hospital

**Neurosurgery Department**

**Head Injury Research Ethics Questionnaire**

Every year approximately 1.4 million people go to A&E with a recent head injury. Most people who have minor head injury recover within three months however, in severe head injuries recovery takes longer and may not be complete

Current treatment of head injury involves supporting the patient while they get better but there is nothing that can be done to actively make the brain get better. Part of this reason is because there is a lot that we don’t know about what happens in head injury.

This is the subject of a lot of ongoing research which we already do at the Royal London Hospital, and the university that we are attached to, Queen Mary University of London.

We are planning a project where we collect samples from patients with head injuries. Any research project needs ethical approval and part of that involves talking to patients. We would like to ask your opinion about this proposed research project.

During acute treatment of head injury, many patients have a pressure monitor inserted into the brain. This usually stays for a few days and is then removed. While the monitor is in the brain, a few brain cells stick to the monitor. The monitor is thrown away after use and any cells that are stuck to it are also thrown away.

Would you consent to the use of the cells that are stuck to the minitor, normally thrown away, for the purposes of research into brain injury?

Yes

No

One of the methods used to reduce pressure inside the brain after a head injury involves inserting a tube to drain excess fluid from the brain. This fluid usually drains continually for several days and is thrown away.

Would you consent to us using this fluid for research purposes?

Yes

No

When the tube is taken out, we would like to use any brain cells that have stuck to the tube for research. Would you consent to us using this for research?

Yes

No

As part of normal care looking after patients with a bad head injury, blood tests are performed every day. Would you consent to us taking extra blood samples for research purposes?

Yes

No

In the future, research Doctors may want to insert a special cannula (tube) into a vein in the neck. This gives us the opportunity to take samples of blood as it leaves the brain, before it mixes with the rest of the blood circulation.

Most patients have a tube inserted into the neck to help with monitoring them on intensive care. This only happens when they are unconscious. Would you consent to having an extra tube inserted into the neck?

Yes

No

During operations it is often necessary for blood in the brain to be removed during the procedure. This is otherwise thrown away.

Would you consent to us using this for research purposes?

Yes

No

During operations it is often necessary for brain tissue to be removed during the procedure This is otherwise thrown away.

Would you consent to us using this for research purposes?

Yes

No
