# Supplementary material for: Defining New Research Questions and Protocols in the Field of Traumatic Brain Injury through Public Engagement: Preliminary Results and Review of the Literature
Source: Emerg Med Int. 2019 Oct 31;2019:9101235. doi: 10.1155/2019/9101235 (PMC6875310; doi:10.1155/2019/9101235)
Supplement: Supplementary Materials — Supplementary material 1: pilot survey created to gauge response from head injury patients and their families to establish perceived acceptability to conducting laboratory analysis of neural tissue that may otherwise be discarded, obtaining peripheral blood samples, and for the insertion of jugular bulb catheters in patients with severe traumatic brain injuries. Supplementary material 2: Survey 1 created following feedback from the pilot survey. This contained further details and the response option of “do not know.” This also included new questions regarding obtaining additional tissue, i.e., brain biopsies at the time of insertion of intracranial pressure monitors, and obtaining extra samples of bodily fluids for analysis. Supplementary material 3: Survey 2 created following feedback from Survey 1 to answer questions regarding anonymity, the secure storage of samples in our laboratory, and that no further research investigations would be conducted at follow-up. [file 9101235.f1.zip › 9101235.f1/Survey 2.docx]

**Head Injury Questionnaire**

Thank you for agreeing to complete this questionnaire.

Every year thousands of people lives are changed forever after sustaining a head injury. At present we have many imaging techniques to look at the brain after an injury has occurred which enables us to know that something has happened. However, a vast amount of information is missing and we don’t yet understand what is happening to the brain on a microscopic or cellular level. We would like to research this by taking tiny samples from the brain in people who have sustained a head injury and test them in a laboratory to try and analyse the effects these injuries have on people.

For other parts of the body, for example the heart or the liver, we know there are specific blood tests we can do to see what is happening and monitor the progress, however the brain is unique and unfortunately we cannot do the same kind of tests.

We frequently insert a small probe in to the head allowing us to measure the pressure in the brain when patients are admitted following a severe head injury. We would like to take a very small amount of brain tissue at the same time to examine what is happening at a cellular level. The samples will be stored securely in our laboratory and will be anonymised. The risks involved in collecting this sample are no greater than the risk of inserting the probe in the first place. We will also collect samples from the brain and circulating fluid around the brain that may otherwise be discarded after an operation, and other samples such as blood and urine to test them in a laboratory to gain further information. These will also be anonymised. We would then follow people up by calling them in 3 months, 6 months and 1 year to see how they are getting on, there would be no further research investigations at these points.

We hope that by doing this research we will be able to find a way of helping improve the outcome in people with severe head injuries, and identify specific investigations that can help predict their outcome.

1. During acute treatment of head injury, many patients have a pressure monitor inserted into the brain. This usually stays for a few days and is then removed. While the monitor is in the brain, a few brain cells stick to the monitor. The monitor is thrown away after use and any cells that are stuck to it are also thrown away.

Would you consent to the use of the cells that are stuck to the monitor, normally thrown away, for the purposes of research into brain injury?

Yes

No

Don’t know

2. Would you consent to us taking a brain biopsy (smaller than the size of a grain of rice) at the time of inserting the monitor?

Yes

No

Don’t know

3. One of the methods used to reduce pressure inside the brain after a head injury involves inserting a tube in to the brain or the spine to drain excess fluid from the brain. This fluid usually drains continually for several days and is thrown away.

Would you consent to us using this fluid for research purposes?

Yes

No

Don’t know

4. When the tube is taken out, we would like to use any cells that have stuck to the tube for research.

Would you consent to us using this for research?

Yes

No

Don’t know

5. As part of normal care looking after patients with a bad head injury, blood tests are performed every day.

Would you consent to us taking extra blood samples for research purposes?

Yes

No

Don’t know

6. As part of normal care looking after patients with a bad head injury, urine, saliva, and stool samples may be taken.

Would you consent to us taking extra samples for research purposes?

Yes

No

Don’t know

7. In the future, research Doctors may want to insert a special cannula (tube) into a vein in the neck. This gives us the opportunity to take samples of blood as it leaves the brain, before it mixes with the rest of the blood circulation.

Most patients have a tube inserted into the neck to help with monitoring them on intensive care. This only happens when they are unconscious. Would you consent to having an extra tube inserted into the neck?

Yes

No

Don’t know

8. During operations it is often necessary for blood in the brain to be removed during the procedure. This is otherwise thrown away.

Would you consent to us using this for research purposes?

Yes

No

Don’t know

9. During operations it is sometimes necessary for brain tissue to be removed during the procedure. This is otherwise thrown away.

Would you consent to us using this for research purposes?

Yes

No

Don’t know

10. Do you have any other comments, questions, or concerns?
